# Is there adaptation of the exocrine pancreas in wild animal? The case of the Roe Deer

**DOI:** 10.1186/1746-6148-8-70

**Published:** 2012-05-28

**Authors:** Paul Guilloteau, Francesca Vitari, Valérie Metzinger-Le Meuth, Laurence Le Normand, Véronique Romé, Gérard Savary, Luc Delaby, Cinzia Domeneghini, Jean Morisset

**Affiliations:** 1 INRA, U1341, Nutrition et Adaptations Digestives, Nerveuses et Comportementales, Domaine de la Prise, 35590, Saint Gilles, France; 2 Department of Veterinary Sciences and Technologies for Food Safety, University of Milan, via Trentacoste n.2, I-20134, Milan, Italy; 3 Université Paris 13, UFR SMBH, 74 rue Marcel Cachin, F-93017 Bobigny, and INSERM U1088 MP3C, Rue des Louvels, F-80037, Amiens, France; 4 INRA, UMR 1348, Physiologie, Environnement et Génétique pour l’Animal et les systems d’Elevage (PEGASE), Domaine de la Prise, 35590, Saint Gilles, France; 5 Service de gastroentérologie, Département de Médecine, Faculté de Médecine et des Sciences de la Santé, Université de Sherbrooke, Sherbrooke, Québec, J1H5N4, Canada

**Keywords:** Pancreas, Roe deer, Cattle, Exocrine secretion, CCK receptors

## Abstract

**Background:**

Physiology of the exocrine pancreas has been well studied in domestic and in laboratory animals as well as in humans. However, it remains quite unknown in wildlife mammals. Roe deer and cattle (including calf) belong to different families but have a common ancestor. This work aimed to evaluate in the Roe deer, the adaptation to diet of the exocrine pancreatic functions and regulations related to animal evolution and domestication.

**Results:**

Forty bovine were distributed into 2 groups of animals either fed exclusively with a milk formula (monogastric) or fed a dry feed which allowed for rumen function to develop, they were slaughtered at 150 days of age. The 35 Roe deer were wild animals living in the temperate broadleaf and mixed forests, shot during the hunting season and classified in two groups adult and young. Immediately after death, the pancreas was removed for tissue sample collection and then analyzed. When expressed in relation to body weight, pancreas, pancreatic protein weights and enzyme activities measured were higher in Roe deer than in calf. The 1^st^ original feature is that in Roe deer, the very high content in pancreatic enzymes seems to be related to specific digestive products observed (proline-rich proteins largely secreted in saliva) which bind tannins, reducing their deleterious effects on protein digestion. The high chymotrypsin and elastase II quantities could allow recycling of proline-rich proteins. In contrast, domestication and rearing cattle resulted in simplified diet with well digestible components. The 2^nd^ feature is that in wild animal, both receptor subtypes of the CCK/gastrin family peptides were present in the pancreas as in calf, although CCK-2 receptor subtype was previously identified in higher mammals.

**Conclusions:**

Bovine species could have lost some digestive capabilities (no ingestion of great amounts of tannin-rich plants, capabilities to secrete high amounts of proline-rich proteins) compared with Roe deer species. CCK and gastrin could play an important role in the regulation of pancreatic secretion in Roe deer as in calf. This work, to the best of our knowledge is the first study which compared the Roe deer adaptation to diet with a domesticated animal largely studied.

## Background

Physiology of the exocrine pancreas has been well studied in domestic (dog, pig, sheep, cattle), and in laboratory (rat, mice, guinea pig) animals as well as in humans. However, it remains quite unknown in wildlife mammals because of either a lack of interest or a relative inaccessibility to the gland.

In European countries, the cattle and the Roe deer *(Capreolus capreolus*) are two species with the closest frame of phylogeny as shown by their karyotypes [1,2]. Both animals have a common ancestor at the level of the Ruminant class but they diverge from the Super Families (Taurus and Elaphoidae) and then from the Bovidae and Cervidae families to obtain genus which concerns both the *Bos* and *Capreoleus* species. The Cervidae is a family considered as one of the most recent branches in the Bovidae family [3].

The ruminants include about 150 species and their digestive systems differ in structure functions and in their adaptation regarding feeding behavior in relation to geographic and climatic diversity. For these reasons, they have been divided into three different feeding types. Sheep and cattle belong to the grass and roughage (GR) eaters. Their gastrointestinal tract (GIT) is well adapted to a diet rich in plant fibers fermented efficiently in their rumen. Other ruminants (approximately 40 %), including the Roe deer, adapted a diet rich in easily digestible plant cell contents and were classified as concentrate selectors (CS). Finally, the third group (about 35 %) including the Red deer (*Cervus elaphus*), is an intermediate mixed feeder (IM) with a mixed diet avoiding plant fiber as much as possible. It has been proposed in IM and mostly CS ruminants that the use of reticular groove as a by-pass route allows a large proportion of their diet content to reach the intestine. Once hydrolysed by the pancreatic and intestinal brush-border enzymes, nutrients are available for absorption [4]. In these different ruminant classes, adaptation of the structure and functions of the GIT in response to dietary trait has been studied but description of the digestive enzymes in the different digestive organs is very limited with the exception of those in the domestic ruminants, such as the ovine and bovine species. Over the years, our research interest has been partly focused on the pancreatic exocrine function in the calf (*Bos taurus*) [5,6].

With our experience in the physiology of the ruminant’s GIT and our access to Roe deer, it was decided to perform a comparative study of the digestive pancreas of Roe deer and calf to evaluate their adaptation to diet, simple diet versus changing diet with space and time. Earlier studies in the calf pancreas established the presence of both cholecystokinin (CCK) receptor (CCKR) subtypes, CCKR-1 and CCKR-2 (or CCKR-A and CCKR-B, respectively) [7]. Furthermore, in higher mammals, the CCKR-2 subtype has been reported to be the most expressed [8]. Therefore, we have investigated the two CCK receptor subtypes in the Roe deer pancreas. This study gives us the opportunity to evaluate the phylogeny evolution between a domestic and a wild ruminant and to confirm that dietary adaptation occurs in wild ruminants.

## Methods

### Animal, diet, feeding and experimental design

Treatments and experiments were conducted according to European Union regulations concerning the protection of experimental animals and to the licence for experimentation in animal given by the French Veterinary Services (N° 03014, April 11^th^ 2008). Forty Holstein-Friesian male calves, bought at about 8 days of age, were reared on straw used as bedding, in individual crates. Up to 4 weeks of age, they were muzzled and received a milk formula based on skim milk powder (66 %), whey powder (10 %), tallow (22 %) and starch, mineral and vitamin mixtures (3 %). The chemical composition of this diet was (% of dry matter, DM) 24.6, 21.9, 43.2, 2.5 and 7.8 respectively, for protein, lipid, lactose, starch and minerals. Calves were fed twice daily at 0830 and 1630 h. They were then assigned into two groups (MF and W). Animals from MF-Group (n = 22) continued to receive exclusively the milk formula and remained monogastric until slaughter. They were always muzzled and were fed twice daily except on Sunday where no evening meal was given. The amounts of food distributed at each meal increased with age from 665 to 1,595 g of dry matter (DM) and its concentration increased likewise from 133 to 190 g of DM/kg of milk formula.

The muzzle was removed from the other calves (W-Group, n = 18) and they were gradually weaned between 4 and 9 weeks onto a solid diet; thus becoming a functional ruminant. They were given a concentrate feed, dehydrated fescue and water for ad libitum intake from 4 weeks of age. The chemical composition of concentrate feed (% of DM) was 17.6, 3.0, 51.9, 10.2 and 7.5 respectively, for crude protein, lipid, starch, crude fiber and minerals. The corresponding values for dehydrated fescue were respectively 16.9, 3.1, about 0, 24.4 and 11.0. All animals of the same group (MF or W) were slaughtered on the same day and slaughter ages were chosen so as to obtain similar mean carcass weights in the two groups.

The thirty five Roe deer described in this study were wild animals which lived in the temperate broadleaf and mixed forests (deciduous woodlands) of the Vendée area (France). They were all shot during the hunting season (from October to February). After examination of body size and dentition, they were classified in two groups: adult (age > 1 y, n = 23) and young (age < 8 months, n = 12). In this Vendée area, the diet composition of these ruminant animals in the wild was unknown until it had been studied by Duncan’s group [9-11]. They reported that the leaves of plants and trees constituted the major part of the regimen as well as the seeds and fruits if they were sufficiently abundant. In contrast, the Graminaceae plants were not consumed. Also, their experiments suggested that at least 80-94 % of the vegetative species growing in this area were ingested at least once per season. Among these available plant species, only one to three was significatively selected and represented the main part (22 to 50 %) of the diet. Thus, in all seasons, forbs and brambles were the principal components, with ivy in winter and acorns in autumn in some years. Roe deer are categorized as a “concentrate selector”, i.e. they select diets with high cell contents (soluble sugar in particular) and low fiber levels. Interestingly, Roe deer in natural habitat also selects tannin-rich plants. In these conditions, an example of the partial chemical composition of the diet (% of DM) ingested by an adult Roe deer, was given by Duncan et al [12] : >10.8, > 7.7, < 20.0 and <0.7 respectively for protein, soluble carbohydrates, lignocellulose and silica. On the other hand, young Roe deer (age < 8 months) suckles its mother and is on a pre-weaning stage. Analyses of Roe deer milk composition resulted in (% DM) 16.5, 37.0, 36.5 and 9.5 respectively for protein, lipid, lactose, and minerals [13].

### Biological and statistical analyses

During the rearing period of the calves, samples of milk formula concentrate and dehydrated fescue feed were collected to have a mean sample to determine DM, nitrogen (N), fat and minerals according to previously described methods [14].

The calves were weighed each week during the experimentation and they were slaughtered 16 to 17 h after their last meal (from the previous day). At that time, they were 138 and 159 days old for the animals coming from MF- and W- Groups, respectively. All the Roe deers were shot during the morning of the day of hunting and weighed immediately after death. The entire GIT was removed and the weight of the pancreas was taken.

Three pieces of the pancreatic tissue were carefully collected (about 1 g per sample), frozen in liquid nitrogen and stored at −20 °C for total protein, enzyme activity and CCK receptor analyses. For protein and enzyme assays, they were homogenized in cold distilled water (1 g of tissue/10 mL of distilled water) and centrifuged for 5 min at 1,000 x g at 4 °C, after thawing of the pancreatic samples. The protein contents were then determined as described by Lowry et al [15]. The activity of trypsin (EC 3.4.21.1) and chymotrypsin (EC 3.4.21.2) were measured using the modified method reported by Lainé et al [16] and the activity of α-amylase (EC 3.2.1.1) was assayed according to Bernfeld [17]. Activities of elastases I and II (EC 3.4.21.36) and lipase (EC 3.1.1.3) were measured using L-alanyl-L-alanyl-L-alanine methyl ester, succinyl-L-alanyl-L-alanyl-L-prolyl-L-leucine-*p*-nitroaninilide and tributyrate as substrates, respectively [18]. The results are expressed as international units (IU) per milligram of pancreatic protein (specific activity) for all the enzymes as well as per kilogram of body weight (BW).

For evaluation by immunohistochemistry of the CCK/gastrin family receptors, fragments of pancreatic tissue were taken from each animal and fixed immediately in 4 % paraformaldehyde in 0.1 M sodium phosphate, pH 7.2 for 24 hours at 4 °C. The tissue samples were then dehydrated in graded ethanol series, cleared with xylene, and embedded in paraffin. Serial microtome sections (4 μm-thick) were obtained and stained with Hematoxilin and Eosin to evaluate morphological structural details. Deparaffinized rehydrated sections were treated with 3 % H_2_O_2_ in distilled water to block the endogenous peroxidase activity. Heat-induced epitope retrieval was performed by heating the slides with sections in a microwave oven in a 0.02-M citrate buffer (pH 6.0) for a total of 10 minutes (two 5-minutes periods at 500 W, with replacement of evaporated buffer). After the slides were cooled at room temperature for 60 minutes, they were rinsed with deionized water and incubated with Normal Goat Serum (DAKO, Italy) diluted at 1:20 for 30 minutes to reduce non-specific background staining. Subsequently, sections were incubated with rabbit (polyclonal) anti-CCKA (or CCK1) receptor (CCKR-A, 1:2000; Abcam, UK) or anti CCKB (or CCK2) receptor antiserum (CCKR-B, 1:2500; Chemicon, USA) in 0.05 M Tris–HCl buffer saline (TBS; 0.05 M, pH 7.4, 0.55 M NaCl) with 1 % bovine serum albumin for 24 hours at 4 ° C. Antigen–antibody complexes were detected with a peroxidase-conjugated polymer which carries secondary antibody molecules directed against rabbit immunoglobulins (EnVisionTM+, DAKO,) applied for 60 minutes at room temperature. Appropriate washing with TBS was performed between each step and all incubations were carried out in a moist chamber. The reaction products were stained with VECTOR-VIP (Vector Laboratories Inc., USA), counterstained with Mayer’s hematoxylin, dehydrated, and permanently mounted.

The control experiments were performed as follows: i) substitution of the primary antisera by normal goat serum, ii) omission of the primary antiserum and incubation with the secondary antibody alone.

Gel electrophoresis and immunoblotting. This procedure was performed as described by Julien et al [19] with the CCKR-A antibody AR6 generously given by Dr M.L. Kruse, Kiel, Germany. The 9262 CCKR-B receptor antibody was a generous gift from Dr J. Walsh, CURE, Los Angeles, CA. Specificity of each receptor antibody has been previously established by pre-incubation of each antibody with its specific antigen.

The SAS program package was used for the statistical data analysis by means of the General Linear Model procedure, using analysis of variance on repeated measures, with one factor (type of animal) corresponding to the 4 types of animals (Roe deer- adult, Roe deer- young, Ruminant calf, Milk-fed calf), under SAS/STAT® [20]. Data are presented as means and SEM. In all statistical analysis, *P* < 0.05 was taken of significance with the level, but the level *P* < 0.10 retained to indicate a tendency.

## Results

The calves remained clinically healthy during the whole study and their relative growth rate was within normal range (1432 ± 32 and 1143 ± 30 g/d for MF- and W-Groups respectively, *P* < 0.05). After killing and samples collection, the general aspect as well as the GIT clinical appearance of each Roe deer was examined and all of them appeared normal.

### Body and pancreas weights and pancreatic total proteins

Overall, the BW and the pancreas weight of the calves were respectively 9–10 and 6–8 folds higher than those of the adult Roe deer (*P* < 0.05). Among calves, the BW of the animals in the W-Group was higher than that of the MF-Group (*P* < 0.05). On average, the weight of young Roe deer (weaning stage, < 8 months of age) had reached 67 % of the adult BW; however, their pancreatic weight represented 86 % of that of adult animals. Pancreas weight, expressed as g/kg of BW, was higher in Roe deer (mainly in young animal) than in the milk-fed or ruminant calves (*P* < 0.05) (Table 1).

**Table 1:** Number, age, body weight of animals and characteristics of their pancreas

Species	Roe deer		Cattle	
Stage	Adult	Young	Ruminant calf	Milk-fed calf
Age	> 1 year	< 8 months	159 days	138 days
			(W group)	(MF group)
Number of animals	23	12	18	22
Body weight (BW^**1**^, kg)	22.4 ± 2.4^**†**"^	15.0 ± 1.2^**‡**"^	227.8 ± 1.3^**§**"^	205.5 ± 1.6^"^
Pancreas weight				
- total (g)	26.4 ± 1.3^**†**^	21.3 ± 2.6^**†**^	203.5 ± 1.7^**‡**^	156.7 ± 1.4^**§**^
- g/kg BW^**1**^	1.18 ± 0.01^**†**^	1.54 ± 0.01^**‡**^	0.89 ± 0.01 ^**§**^	0.76 ± 0.01^**§**^
Pancreatic proteins				
- Total (g)	3.7 ± 0.2^**†**^	3.1 ± 0.5^**†**^	31.1 ± 0.3^**‡**^	25.7 ± 0.2^**§**^
- mg/g pancreas	138 ± 2^**†**^	134 ± 1^**†**^	151 ± 1^**‡**^	165 ± 1^**‡**^
- mg/kg BW^**1**^	164 ± 1^**†**^	208 ± 3^**‡**^	134 ± 4^**§**^	125 ± 5^**§**^

Values are means ± SEM (standard error of the mean).

^**†, ‡, §**^^**, "**^: Means within a row with different superscripts differ (*P* < 0.05).

^**1**^ BW = Body weight.

In each species, total pancreatic proteins (mg/pancreas) were higher in ruminant animals than in pre-weaning (NS) or milk-fed (*P* < 0.05) animals, respectively. This difference disappeared however when protein values were expressed as mg/g pancreas or as mg/kg BW, but with this latter expression, the highest value was obtained in young Roe deer group (Table 1). Proteins were always lower in Roe deer than in calf pancreas (*P* < 0.05) except when expressed as mg/kg BW.

### Pancreatic enzyme activities

As shown in Table 2, except for lipase, specific activities of each pancreatic enzyme were always greater or similar in Roe deer than in the calf and more so for chymotrypsin and elastase II (*P* < 0.001), with 5 to 8-fold increases. With the exception of chymotrypsin values which show a significant increase of 39 % (*P* < 0.05) in the adult, there was no difference between young and adult Roe deer for all the other enzymes. In calves, values were significantly higher (*P* < 0.05) in the W- group when compared to the MF- group only for trypsin and amylase.

**Table 2:** **Specific activities (IU**^**1**^**/mg proteins) of the pancreatic enzymes**

Species	Roe deer			Cattle		
Stage	Adult	Young		Ruminant calf	Milk-fed calf	
Age	> 1 year	< 1 year	%Δ^1^	159 days	138 days	%Δ^2^
				(W group)	(MF group)	
Chymotrypsin	1936 ± 33^**†**^	1397 ± 65^**‡**^	39.0	12 ± 43^**§**^	16 ± 35^**§**^	−25.0
Trypsin	95 ± 2^**†**^	90 ± 3^**†‡**^	5.5	102 ± 2^**†**^	76 ± 1^**‡**^	34.2
Elastase I	279 ± 7^**†**^	233 ± 12^**†‡**^	20.0	160 ± 35^**‡§**^	57 ± 55^**§**^	180.7
Elastase II	1423 ± 28^**†**^	1286 ± 50^**†**^	10.7	270 ± 139^**‡**^	194 ± 219^**‡**^	39.2
Lipase	209 ± 70^**†**^	237 ±34^**†**^	−11.9	477 ± 16^**‡**^	738 ± 27^**‡**^	−35.4
Amylase	15.6 ± 0.2^**†§**^	14.1 ± 0.3^**†**^	10.6	16.2 ± 0.4^**§**^	8.9 ± 0.3^**‡**^	81.4
Ratio:						
Chymotrypsin/Trysin	22.4 ± 0.3^**†**^	15.4 ± 0.7^**‡**^	45.5	0.1 ± 0.4^**§**^	0.3 ± 0.3^**§**^	−66,7

Values are mean ± SEM (standard error of the mean).

^**†, ‡, §**^ Means within a row with different superscripts differ (*P* < 0.05).

Effect of species: Means within a row are different between species (P < 0.001) for chymotrypsin and Elastase II.

IU^**1**^: International Unit.

%Δ: Difference between values: Adult values-Young values (Δ^1^) or Ruminant values- Milk-fed values (Δ^2^) expressed as % of “Young values” and “Milk-fed values” respectively.

**Figure 1:**
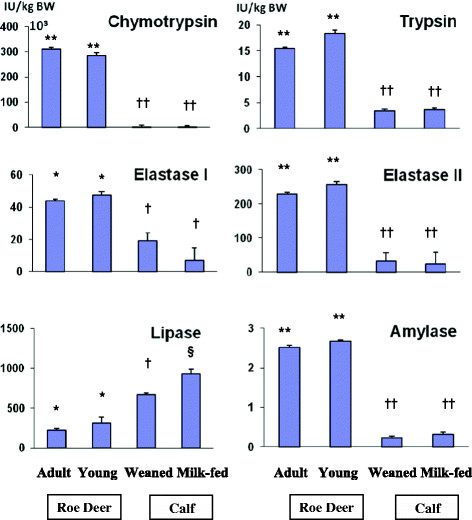
**Pancreatic enzyme activities relative to body weight in Roe Deer and cattle, in relation with their nutritional conditions**. Values are mean ± SEM (standard error of the mean). BW: Body weight, IU: International Unit. *^, †, ‡^ For each enzyme, values with different superscripts differ (*P* < 0.05). **^, ††, ‡‡^ For each enzyme, values with different superscripts differ (*P* < 0.001).

When enzyme activities are expressed in relation to BW, the difference between species is much more evident, as shown in Figure 1. In this case, trypsin, elastase II, amylase and mainly chymotrypsin were the most affected with changes of 5 to 150-fold (*P* <0.001). In bovine (except for lipase) and in Roe deer, there was no differences between enzymatic activities for each enzyme between the ruminant animal groups and pre-weaning or milk-fed animal groups, respectively.

### CCK/gastrin family receptors – immunohistochemistry and western-blot

**Figure 2:**
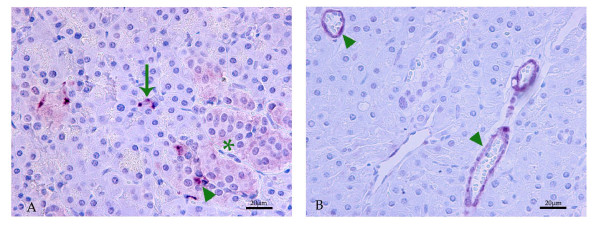
**Cellular localization of CCK receptor subtype 1 (CCKR-1, A) and CCK receptor subtype 2 (CCKR-2, B) proteins on pancreas section of an adult Roe deer, by immuno-histochemical analysis**. **A**- CCKR-1 immuno-positivity was present: in the cytoplasm and cell membrane of endocrine cells belonging to the pancreatic islets (asterisk) and particularly in the cytoplasm of some cells (arrow head); in the cytoplasm of endocrine cells that were localized among pancreatic exocrine cells (arrow). **B**- CCKR-2 immuno-positivity was detected in the cytoplasm of myocytes, of arterial blood vessels (arrow head).

**Figure 3:**
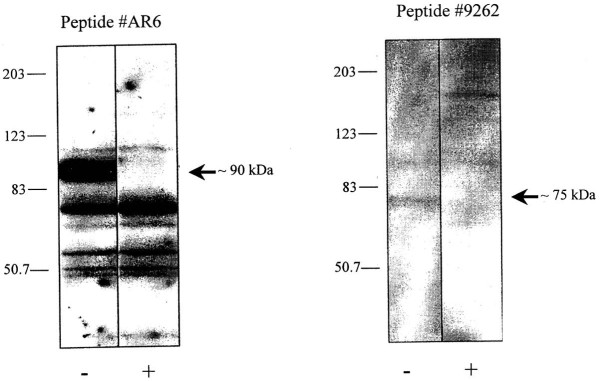
**Roe deer pancreas homogenates (40 μg protein) were used for Western blotting analysis with antibodies AR6 (1:1,000) and 9262 (1:10,000)**. Specificity was established by pre-incubation of each primary antibody for 2 h at room temperature with 40 μg of the corresponding peptide antigen

In both the Roe deer adult and young animals, CCKR-1 immuno-histochemistry stained insular endocrine cells primarily belonging to the pancreatic islets (Figure 2A, asterisk); some cells were more intensively labeled than others (Figure 2A, arrow head). Furthermore, immunoreactive endocrine cells were identified within the exocrine parenchyma (Figure 2A, arrow). No immunoreactivity was detected in exocrine cells, fibroblasts or nerve fibers of the Roe deer pancreas. CCKR-2 immunopositivity was observed at all ages in the tunica intima and of several arterioles but not in capillaries located in the pancreatic parenchyma (Figure 2B, arrow heads). As illustrated in Figure 3, the adult Roe deer pancreas homogenate contains the CCKR-1 protein as a 90 kDa protein. Specificity of the selected AR6 antibody is confirmed by its pre-incubation with an excess of peptide antigen (+). On this same homogenate preparation, we have also detected the CCKR-2 protein as a 75 to 80 kDa protein using the specific 9262 antibody. Specificity of this 9262 antibody was also established after its pre-incubation with its specific antigen (+). The relative abundance of this receptor is much less important than that of the CCKR-1 based on the facts that both gels were run on the same day with an equal amount of proteins applied on the gel.

## Discussion

Roe deer and cattle (including calf) belong to Cervidae and Bovidae families respectively, both are members of the Ruminantia suborder. One aim of this work was to evaluate in Roe deer the adaptation to diet in relation with animal domestication. Thus we have characterized the pancreas of the Roe deer as well as its content in pancreatic enzymes to compare data obtained in Roe deer with those collected in calf. When data are expressed in relation to BW, the pancreas and pancreatic protein weights are higher in Roe deer than in calf as well as most enzyme activities measured. Moreover, both receptor subtypes of the CCK/gastrin family peptides are present in the Roe deer’s pancreas like in the calf’s. The consequences for digestive processes and their regulation are suggested in the following discussion.

### GIT morphology and physiology

Pancreas weight was higher in ruminant animals than in weaning or milk-fed animals, as previously shown in calves and sheep [6]. This difference however disappeared when the organ weight was expressed as a percentage of BW in calves but not in Roe deer. In milk-fed and in weaned calves aged from 138 to 156 d, the pancreas weight expressed as percentage of BW is similar to the value obtained in adult (0.78 g/kg BW, [21]). In contrast, for the young Roe deer, this value was higher than in adult. To explain these differences, it would be interesting to have histometrical data (number of cells per acinus, cell area, etc.) to determine whether these variations come from pancreas structure and/or higher secretory capacities.

Compared with ruminant calves, the GIT of young (weaning) and adult (ruminant) Roe deer is characterized by several particularities. Roe deer have a selective feeding behaviour with a high proportion of soluble plant cell contents in their diet [10,11]. Moreover, a number of morphological and physiological particularities of their GIT were underlined; they exhibit a relatively small forestomach with large orifices between the different sections, a short retention time of ingesta, relatively large salivary glands producing high volumes of saliva, and a highly developed reticular groove which is retained in the adults [4,22,23]. Considering these digestive particularities, we can speculate that Roe deer have incomplete fermentation in their rumen, a ruminal escape of digesta components (starch, fats, etc.) and/or rumen bypass of rapidly digestible components (soluble sugars, etc.) from the oesophagus to the abomasum down to the reticular groove. These anatomical and physiological observations suggest that a part of their diet components can reach the abomasum and then the intestinal lumen and be digested according to the mechanisms described in monogastric animals. These observations justify that milk-fed calves be introduced in our experiments since in the GIT of this animal, milk bypasses the rumen thanks to the reticular groove closure, as it is the case for a part of the Roe deer’s diet. In such cases, enzymatic digestion plays a much more important role than in ruminant calves.

Another morphological and physiological particularity of the GIT compared with calf is that Roe deer have a shorter intestine (relative to BW) and a smaller ratio between small and large intestine (70:30 vs 82:18) [24]. In these conditions, even the digestion is important in the small intestine in both animals, that of the Roe deer could be less effective, at least for starch and sugars, due to less intestinal enzymes and a shorter transit time. But the enzyme activities measured in the pancreas were much greater in Roe deer than in calf and this could compensate this intestinal deficiency in enzymatic digestion.

### Enzymes activities

In Roe deer, pancreatic enzyme activities were seldomly studied and references on this subject are very scarce. In this study, we have compared Roe deer and calf digestive enzymes also because of available data on domestic ruminant animals [5,6]. In Roe deer, Rowell-Schäfer et al [23] reported, we believe, the only data available on pancreatic α-amylase in comparison with those measured in sheep. In agreement with our data, they showed that α-amylase activity was about twice that found in the domestic ruminant animal. They also investigated intestinal disaccharidase activities and found them higher in Roe deer than in ruminant sheep and goats. In the current experiment, with regards to lipase in calf, it seems that there was an adaptation to lipid level since lipid level in diet ingested by Milk-fed animals was higher than in solid diet consumed by weaned calves. By contrast there was no adaptation in the Roe deer species. Also, this relation did not stand true for proteolytic enzyme activities and protein levels in the diets.

Thus, proteolytic enzyme activities were largely higher in Roe deer than in calves, mainly for chymotrypsin and elastase II. This observation could be in relation to particular digestive products observed in Roe deer. Among these products, Roe deer secrete about 9–14 times more saliva (6.7-13.5 vs 0.5-1.5 ml/10 min/kg BW) than sheep [22]. Saliva usually contains high amounts of proline-rich proteins (PRPs) whereas their levels are relatively low in calf and sheep saliva. Furthermore, pancreatic chymotrypsin contents in adult Roe deer are 150-fold higher than in ruminant calf. Of interest is the fact that chymotrypsin cleaves the amino-acid bonds in proteins where proline is present. Moreover, elastase II specifically hydrolyses peptide bonds involving highly hydrophobic amino acids [25] which are abundant (30 %) in the first part of the primary structure of PRPs [26]. These phenomena could allow the Roe deer to reuse its PRPs.

Interestingly, the Roe deer prefer tannin-rich plants which provides a nutritional benefit for the animal [11,27]. When present in diets of ruminants, tannins generally reduce growth performance and increase the excretion of nutrients in ruminants. Moreover, in human diets and in those of non-ruminant animal species, tannins can reduce the digestibility of proteins, carbohydrates and minerals; they can also result in lower digestive enzyme activities and cause damage to the mucosa of the GIT [28]. By contrast, the presence of tannins in the diet at a low dose could result in the formation of tannin-protein complexes which can enhance protein assimilation by avoiding microbial breakdown of protein in the rumen. Because PRPs bind tannins, they thus reduce their deleterious effects on protein digestion in Roe deer for which the presence of tannins in the diet is considered to have benefit effects.

In bovine species, the specific enzymatic activities of trypsin, elastase 1 and amylase were higher in ruminant than in milk-fed calves; these changes illustrate the modifications in their diet composition [29]. In contrast, there was no modification in the corresponding values for chymotrypsin, elastase II and lipase. However, in milk-fed calves, lipid intake declines after weaning. In Roe deer, few differences were observed between young and adult animals in their pancreatic enzymes with only chymotrypsin specific activity being much lower in young Roe deer than in adults. Even if these young animals may have been at least partially ruminants, this phenomenon could be linked to a lesser ingestion of tannin-rich plants and consequently to a lesser production of PRPs in saliva in the young.

### CCK/gastrin family receptors

Until now and to the best of our knowledge, no data have been reported on the gut regulatory peptides and more particularly those on the CCK/gastrin family, as well as their receptors in Roe deer pancreas [8]. In this study, the immunohistochemical findings illustrate, for the first time, the presence of both CCK-1 and CCK-2 receptor subtypes in the Roe deer pancreas. These results are well supported by the western blot presented which also clearly indicates the presence of both subtypes in tissue homogenates. The molecular weights of both subtypes, around 90 kDa for the CCKR-1 and 75–80 kDa for the CCKR-2 agrees with those previously found in rat pancreatic cell membranes (CCKR-1) and in human and rat pancreatic homogenates (CCKR-2) [19,30]. The differences in molecular weight between species may depend on the glycolysation status of each protein. These two receptors have different affinities for CCK and gastrin. The CCKR-1 subtype exhibits a high affinity for sulphated CCK and a 1,000-fold lower affinity for gastrin, whereas the CCKR-2 subtype interacts with gastrin and CCK with almost the same affinity and poorly discriminates sulphated and non-sulfated peptides [31]. Even though we cannot compare hormone data between species in this study, we can ascertain the presence in the pancreas of both types of CCK receptors. This suggests that the corresponding regulatory peptides could act in the regulation of the development and production of the Roe deer pancreas as well as their secretory response to hormone stimulation.

The CCKR-1 has been localized in the pancreas β- and α-cells of several species, like the mouse, rat, human and pig [30] and the calf [7]. The presence of the CCKR-1 in islet cells of Roe deer pancreas suggests a direct action of CCK on the regulation of islet cell secretion. This effect has been suggested by others scientists [30,32] who localized CCKR-1 in islet cells. In other species, CCKR-1 was also localised in pancreatic endocrine cells out of islet. In the rat pancreas, immunofluorescence confocal microscopy studies and *in situ* hybridization studies localised CCKR-1 in both insulin- and glucagon-producing cells. In pig and human pancreas, immunofluorescence and confocal microscopy studies support the expression of CCKR-1 in glucagon secreting cells only [33]. In Roe deer, we have observed CCKR-1 in endocrine cells but not in acinar cells; therefore, we can suggest that in this species CCK may also play an indirect role on the exocrine pancreas, like in pig and rat. For instance, some authors [34,35] have suggested that in the pig, CCK could indirectly stimulate the exocrine pancreas by a mechanism mediated locally in the small intestine. Furthermore, the cytoplasmic processes of endocrine cells found positive for CCKR-1 were in close contact with acinar cells. This particular morphological feature suggests that these endocrine cells may control the functions of other cells *via* paracrine secretion consequently to CCK stimulation.

Morisset et al. [30] have demonstrated the localization of CCKR-2 protein in the islet’s δ-cells which secrete somatostatin and its unique presence cells of the calf and cow. However, Saillan-Barreau et al [36] reported the major site of CCKR-2 expression in glucagon cells in adult and fetal human pancreas. This does not occur in pancreas of Roe deer, since CCKR-2 were only detected in the tunica intimae of several arterioles, suggesting a possible important role of CCK upon the regulation of blood flow in pancreas. Indeed, in Sprague–Dawley rats, CCK mediated vasodilatation of the mesenteric vascular bed *via* its pre-synaptic CCKR-2 release [37]. Similarly caerulein, a CCK analogue, was shown to increase pancreatic blood flow and vascular conductance in rat *via* activation of CCKR-2 receptors [38].

## Conclusions

In conclusion and under our experimental conditions, while we take into account the pancreas and pancreatic protein content relative to body weight, as well as chymotrypsin, trypsin and elastase 2 activities, it seems that the values obtained in young animal reach that measured in weaned animals earlier in bovine species than in Roe deer species. For these same parameters but also for the other enzymes measured (except for lipase), the quantities are largely lower in calf than in Roe deer. Simultaneously, domestication and rearing cattle resulted in ingestion of simplified diets with components well digestible. In contrast, wild animal like Roe deer belongs to the typical concentrate selector species which have diversified food in the nature but with an adapted consumption to its GIT characteristics with plant consumption in the absence of post-ingestive consequences unfavourable to its well-being. Overall and to extend the results that we have obtained, we suggest that bovine species could have lost some digestive possibilities (no ingestion of great amounts of tannin-rich plants, ability to secrete high amounts of PRPs, etc.) compared with Roe deer species. Moreover, we have demonstrated that CCK-2 receptor subtype is present in pancreas of wild animal while these receptors were previously identified only in superior species. The detection of an expression of CCK-1 receptor subtype in islet cells of the endocrine Roe deer pancreas implies a possible CCK regulation of islets hormone secretion. The detection of CCK-2 receptor subtype in pancreatic arterioles, suggests that gastrin and CCK could play a role in the regulation of pancreatic blood flow. These changes in digestive strategies related to species evolution (natural or dependent on human intervention) could be a suitable approach to better understand the adaptation of digestive tract development and its regulation.

## Abbreviations

BW: Body weight; CCK: Cholecystokinin; CCKR: Cholecystokinin receptor; CCKR-1 (or CCKR-A): Cholecystokinin receptor subtype 1; CCK-2 (or CCKR-B): Cholecystokinin receptor subtype 2; CS: Concentrate selector; DM: Dry matter; GIT: Gastrointestinal tract; GR: Grass and roughage; IM: intermediate mixed feeder; IU: International unit; MF-group: Milk-fed group; N: Nitrogen; PRPs: Proline-rich proteins; W-group: Weaned group.

## Competing interests

The authors declare that they have no competing interests'.

## Authors’ contributions

PG conceived the study, put in place its design, was responsible for the coordination, and drafted the manuscript. LLN, VR and GS participated to samples collection, enzymatic analyses and data collection. FV and CD carried out the immunohistochemistry analysis while JM executed the gel electrophoresis and immunoblotting. LD participated to the design of the study and performed the statistical analysis. VMLM participated to the design of the study and (with JM) to draft the manuscript. All authors participated to the data interpretation, red and approved the final manuscript.
